# Tactile learning transfer from the hand to the face but not to the forearm implies a special hand-face relationship

**DOI:** 10.1038/s41598-018-30183-5

**Published:** 2018-08-06

**Authors:** Dollyane Muret, Hubert R. Dinse

**Affiliations:** 1 Neural Plasticity Laboratory, Institute for Neuroinformatics, Ruhr-University, 44801 Bochum, Germany; 2 Department of Neurology, BG University Hospital Bergmannsheil, 44789 Bochum, Germany

## Abstract

In the primary somatosensory cortex, large-scale cortical and perceptual changes have been demonstrated following input deprivation. Recently, we found that the cortical and perceptual changes induced by repetitive somatosensory stimulation (RSS) at a finger transfer to the face. However, whether such cross-border changes are specific to the face remains elusive. Here, we investigated whether RSS-induced acuity changes at the finger can also transfer to the forearm, which is the body part represented on the other side of the hand representation. Our results confirmed the transfer of tactile learning from the stimulated finger to the lip, but no significant changes were observed at the forearm. A second experiment revealed that the same regions on the forearm exhibited improved tactile acuity when RSS was applied there, excluding the possibility of low plastic ability at the arm representation. This provides also the first evidence that RSS can be efficient on body parts other than the hand. These results suggest that RSS-induced tactile learning transfers preferentially from the hand to the face rather than to the forearm. This specificity could arise from a stronger functional connectivity between the cortical hand and face representations, reflecting a fundamental coupling between these body parts.

## Introduction

It is not well-understood how cortical plasticity translates into perceptual abilities and what are its limits. Due to their clear topographic organization and their role in sensory processing, primary sensory cortices are particularly appropriate for investigating the spatial limits and the perceptual consequences of plastic changes. Within the primary somatosensory cortex (SI), large-scale plastic changes have been predominantly observed across the functional border separating the hand and the face representations, which represents one of the major discontinuities in the so-called *homunculus* described by Penfield and Boldrey in 1937^1^. These changes were typically reported following massive deprivation of tactile inputs to the hand due to amputation^2^, deafferentation^3^, or anaesthesia^4,5^. Thus, it has been suggested that hand-face cross-border plasticity arises from competitive mechanisms between neighbouring areas striving for cortical territory. Extending this framework, we recently demonstrated that cross-border plastic changes from the finger to the face can be induced in SI following an “exposure-based” repetitive stimulation protocol^6^. This protocol, referred to as repetitive somatosensory stimulation (RSS) or training-independent learning^7^, was repeatedly proven to induce plastic changes within the cortical representation of the hand in SI that were specific to the site of stimulation and associated with an increased tactile acuity at the stimulated finger^8–11^. Our recent findings additionally showed that the plasticity induced in the face representation following RSS of the finger is also associated with an increased tactile acuity at the face^6,12^.

However, it remains elusive whether such cross-border changes are specific and limited to the face, suggesting a special functional relationship between the hand and the face, or whether they are related to the cortical proximity of the representations. The latter is supported by the SI plasticity literature, which shows that depriving the hand from its inputs (via deafferentation or amputation) can also lead to plastic changes^3^ and referred sensations^13^ across the hand-arm boundary, which is located on the opposite side of the hand representation within the *homunculus*. Therefore, considering the hand as a single functional unit, one could expect the plastic changes induced by RSS at the finger to also affect the arm representation. Accordingly, perceptual changes could also transfer to the arm, supporting the idea that deprivation- and learning-related plastic processes share similar mechanisms. On the other hand, the work of Graziano and colleagues (see^14^) highlighted the importance of functional associations and synergy between body parts. From that perspective, one could expect the plastic and behavioural changes to be facilitated between the hand and the face compared to between the hand and the arm, due to the higher probability of tactile co-activation between the hand and the face in every day behaviour. To test this hypothesis, we decided to investigate the possibility of changes at the forearm, which was previously found to be involved in cross-border plastic changes, and which displays also a low functional association with the hand at the tactile level. We used a high-frequency RSS protocol^15^ to induce plastic and perceptual changes at a given body part. To explore the pattern of perceptual transfer, we first assessed the tactile acuity of healthy participants at both index fingers (right-D2 and left-D2), the right forearm (right-fArm), and above the right upper lip (right-Lip) before and after RSS of the right-D2. In a second experiment, to test whether the right-fArm is susceptible to RSS-induced changes, we assessed the tactile acuity of healthy participants at the right-fArm before and after RSS of the same region.

## Results

### Experiment 1

**Figure 1:**
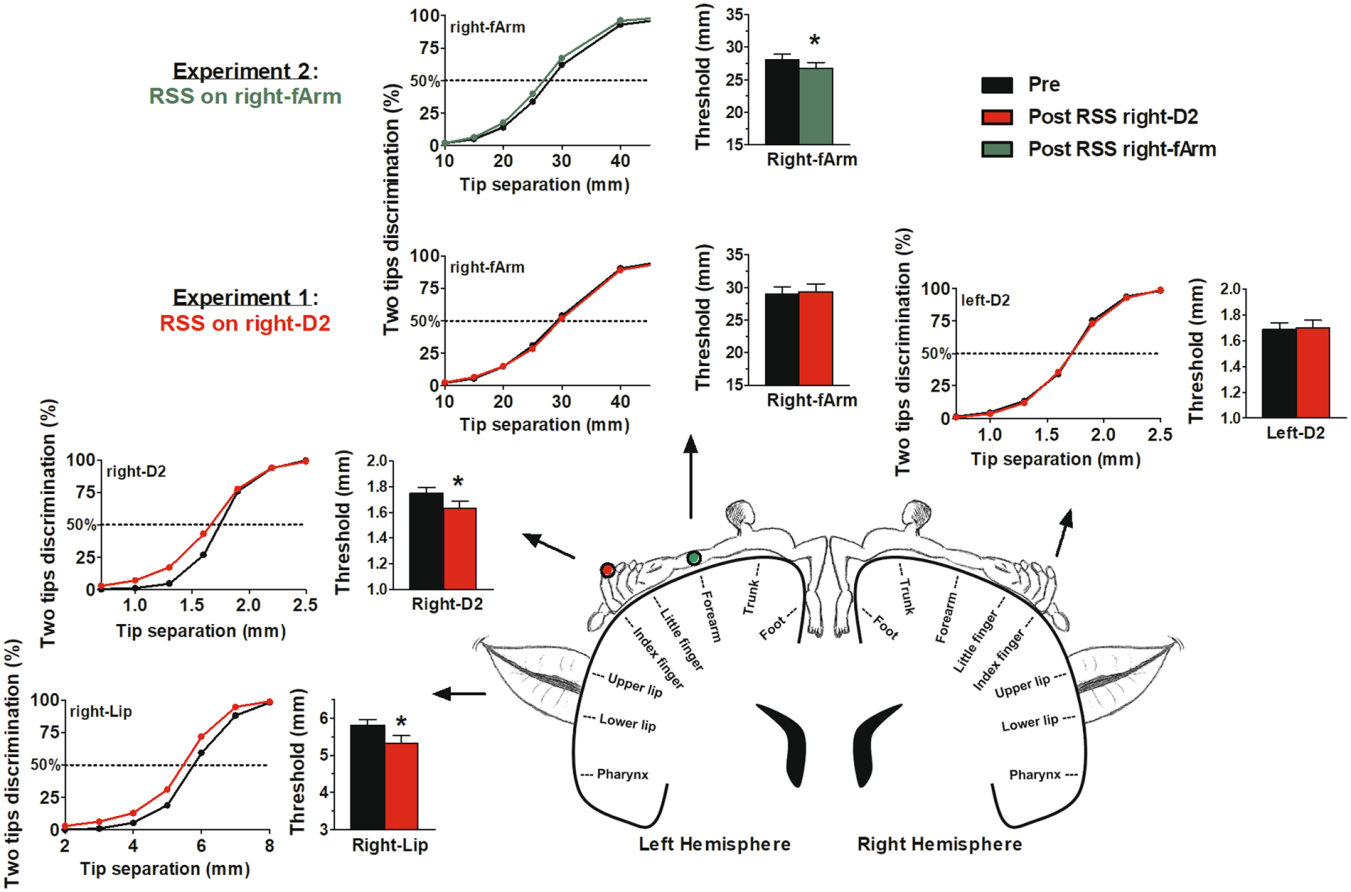
Improvement in tactile acuity transfers from the finger to the face but not to the forearm. Bar plots: mean two-point discrimination threshold pre- (black) and post-RSS applied to the right-D2 (red) or to the right-fArm (green). Thresholds were assessed at the right- and left-D2, the right-Lip, and the right-fArm (mean ± SEM). *p < 0.01 (five paired t-tests, with Bonferroni correction). Psychometric curves: mean psychometric curves. The horizontal dashed line shows the threshold level.

For each of the four tested regions, thresholds, d-prime values, and response criteria were stable across the two baseline sessions (see Table 1). After 40 min of RSS applied to the right-D2, the thresholds significantly decreased at the stimulated right-D2 (*t*_(17)_ = 3.683, *p* = 0.002 < *p*_*Bonf*_, Fig. 1, red plots), but not at the left-D2 (*t*_(17)_ = −0.299, *p* = 0.768, Fig. 1). In addition, while a similar threshold decrease was found for the right-Lip (*t*_(17)_ = 3.933, *p* = 0.001 < *p*_*Bonf*_, Fig. 1), no significant threshold changes were observed at the right-fArm (*t*_(17)_ = −0.512, *p* = 0.615, Fig. 1).

**Table 1:** Baseline stability.

	Body part	Thresholds (mm)				D-prime				Response criteria			
		S1 (mean ± SD)	S2 (mean ± SD)	Paired t-test		S1 (mean ± SD)	S2 (mean ± SD)	Paired t-test		S1 (mean ± SD)	S2 (mean ± SD)	Paired t-test	
				*t*	*p*			*t*	*p*			*t*	*p*
Exp1 (n = 18)	right-D2	1.77 ± 0.22	1.72 ± 0.16	1.065	0.302	0.93 ± 0.29	1.01 ± 0.20	−1.236	0.233	0.60 ± 0.11	0.63 ± 0.04	−1.418	0.174
	left-D2	1.73 ± 0.23	1.65 ± 0.23	1.520	0.147	1.00 ± 0.28	1.08 ± 0.28	−1.454	0.164	0.61 ± 0.07	0.62 ± 0.04	−0.490	0.630
	right-Lip	5.86 ± 0.68	5.73 ± 0.75	1.222	0.238	0.82 ± 0.28	0.88 ± 0.30	−1.559	0.137	0.57 ± 0.13	0.58 ± 0.11	−0.850	0.407
	right-fArm	28.48 ± 4.31	29.54 ± 5.33	−1.350	0.195	0.96 ± 0.27	0.90 ± 0.33	1.093	0.290	0.61 ± 0.05	0.57 ± 0.11	1.336	0.199
Exp2 (n = 16)	right-fArm	28.21 ± 3.96	27.94 ± 3.12	0.355	0.728	0.95 ± 0.26	0.99 ± 0.19	−0.889	0.388	0.60 ± 0.12	0.63 ± 0.03	−1.240	0.234

Mean two-point discrimination thresholds, d-prime values, and response criteria of participants for the two baselines (S1 and S2) in each experiment (1 and 2) and for each tested body part. Paired t-tests revealed no significant differences between the two baselines.

D-prime analysis confirmed a significant gain in sensitivity after RSS for the right-D2 (*t*_(17)_ = −3.822, *p* = 0.001 < *p*_*Bonf*_) and right-Lip (*t*_(17)_ = −4.275, *p* = 0.001 < *p*_*Bonf*_); while, d-prime values remained stable for the left-D2 (*t*_(17)_ = 0.379, *p* = 0.709) and right-fArm (*t*_(17)_ = 0.027, *p* = 0.979). Conversely, the response criteria of participants remained stable for all tested regions (right-D2: *t*_(17)_ = −0.298, *p* = 0.770; left-D2: *t*_(17)_ = 1.022, *p* = 0.321; right-Lip: *t*_(17)_ = −1.357, *p* = 0.192; right-fArm: *t*_(17)_ = −0.337, *p* = 0.740).

**Figure 2:**
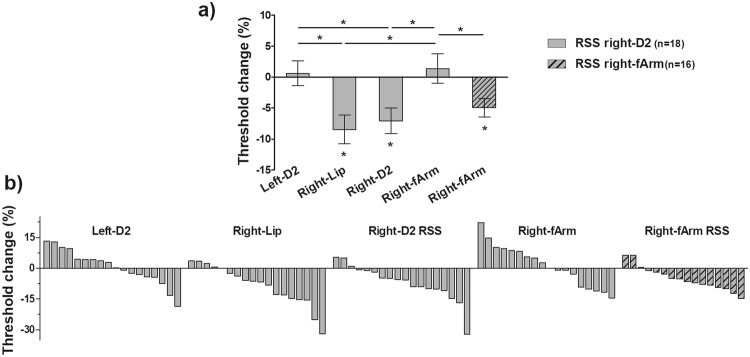
RSS-induced threshold changes observed at the right-D2 and right-Lip showed similar average effect sizes, and were observed in the majority of individuals. (**a**) Mean two-point discrimination threshold changes observed after RSS of the right-D2 (solid bars) at the left-D2, right-D2, right-fArm, and right-Lip (mean ± SEM), and after RSS of the right-fArm (hatched bars) at the right-fArm. Changes are expressed as percentage of the threshold measured before RSS. *p < 0.01 (five t-tests against zero, with Bonferroni correction). *p < 0.05 (repeated measure ANOVA with Bonferroni post-hoc analysis). When comparing the threshold changes obtained at the right-fArm in both experiments, an independent t-test was used (*p < 0.05). (**b**) Percent of threshold changes observed after RSS of the right-D2 (solid bars) or right-fArm (hatched bars) at each tested region for each individual. Note that values are rank ordered, so a given participant is not represented in the same position across graphs.

To compare RSS-induced changes across the different regions, threshold changes were calculated and expressed as a percentage of the threshold obtained before RSS. After RSS of the right-D2, thresholds significantly decreased by 7.06% (±2.06, *t*_(17)_ = −3.429, *p* = 0.003 < *p*_*Bonf*_) at the right-D2 and by 8.46% (±2.31, *t*_(17)_ = −3.663, *p* = 0.002 < *p*_*Bonf*_) at the right-Lip (Fig. 2a), while thresholds changes at the left-D2 and right-fArm did not differ significantly from zero (left-D2: *t*_(17)_ = 0.299, *p* = 0.769; right-fArm: *t*_(17)_ = 0.575, *p* = 0.573, Fig. 2a). A repeated measures ANOVA revealed that threshold changes significantly differed between regions (*F*_(1,17)_ = 6.962, *p* = 0.001). Post-hoc analysis showed that threshold changes obtained at the right-D2 and right-Lip differed significantly from those obtained at the left-D2 (*p* = 0.042 and *p* = 0.010, respectively) and right-fArm (*p* = 0.019 and *p* = 0.004, respectively). Conversely, no significant difference was found between threshold changes observed at the right-D2 and right-Lip (*p* = 0.999).

While no linear correlation was found between threshold changes observed at the right-D2 and right-Lip, the distribution of threshold changes across individuals (Fig. 2b) confirmed the consistency of the RSS-induced changes since the majority of individuals exhibited threshold decreases at the right-D2 and right-Lip (see also Supplementary Table S1 and Fig. S1 for individual data).

### Experiment 2

Since no significant changes were observed for the right-fArm following stimulation of the finger, a second experiment was conducted to test whether the stimulation was effective enough to drive changes at the forearm level at all. We assessed the tactile acuity of healthy participants at the right-fArm before and after RSS of this forearm. As in Experiment 1, thresholds, d-prime values, and response criteria were stable across the two baseline sessions (see Table 1). Following 40 min of stimulation of the right-fArm, thresholds significantly decreased from 28.07 ± 0.81 mm (mean ± SEM) before RSS to 26.70 ± 0.95 (mean ± SEM) after RSS (*t*_(15)_ = 3.042, *p* = 0.008 < *p*_*Bonf*_, Fig. 1, green plots). The d-prime analysis confirmed a significant gain in sensitivity after RSS (*t*_(15)_ = −4.229, *p* = 0.001 < *p*_*Bonf*_); while, the response criteria remained stable (*t*_(15)_ = −1.251, *p* = 0.230).

An independent t-test revealed the right-fArm threshold changes obtained in Experiment 2 significantly differed from those obtained in Experiment 1 (*t*_(32)_ = 2.192, *p* = 0.036, Fig. 2a). Despite the slightly smaller size effect (0.39) compared with the right-D2 (0.57) and right-Lip (0.62), the distribution of threshold changes across individuals (Fig. 2b) revealed that the majority of individuals exhibited threshold decreases on the forearm after its stimulation (see also Supplementary Table S2 and Fig. S2 for individual data).

## Discussion

Previous reports found that RSS applied to a finger induced perceptual improvement across the hand-face border^6,12^. To better understand the mechanisms involved and the specificity of this transfer of learning, we investigated whether similar perceptual changes can also be observed at the forearm level, which is cortically represented on the other side of the hand representation and displays a low tactile association with the hand. While our results confirmed the transfer of tactile learning from the stimulated finger to the lip, no significant changes in tactile perception were observed at the forearm. These results suggest that training-independent tactile learning transfers preferentially from the finger to the face rather than to the forearm. In addition, the results of the second experiment provide the first evidence that RSS can be applied to other body regions than the fingers and induces similar local perceptual benefits.

The specificity of tactile learning has been repeatedly investigated, but mainly for a given body part, in particular within and across hands. For instance, training-dependent learning at a finger was found to transfer to the adjacent and homologous fingers^16–21^. This is in contrast to the so-called training-independent learning induced by RSS, which is specific to the site of induction within and across hands^15,22^. To the best of our knowledge, the possible transfer of training-dependent learning across body parts has not been investigated yet, considering the hand as a single body part. A study on blind people reading Braille reported improved acuity at the reading fingers but no significant alterations at the lips; although, acuity thresholds were slightly lower than in sighted participants^23^. This observation suggests that there may be different underlying mechanisms for the use-dependent plasticity driven by life-long Braille reading vs. RSS. Thus, our data provide the first evidence that transfer of cross-border changes show a preferred direction from hand to face, rather than from hand to forearm.

To rule out the possibility that the forearm might be more resilient to plastic changes, we conducted a second experiment in which the stimulation was applied to the forearm. Our results provide the first evidence that RSS-induced perceptual changes can be similarly induced at the forearm. The noticeable (but non-significant) smaller effect size at the forearm than at the right-D2 might be attributable to suboptimal stimulation conditions. To the best of our knowledge, these were the first experimental attempts to drive plastic changes at the forearm, which has a low receptor density and, consequently, a small cortical magnification. Although we adapted the spatial scale of the RSS procedure to the individual’s acuity on the forearm, it is more than conceivable that a better optimization of stimulation conditions (in particular, its intensity) would have led to a range of changes more similar to those typically found on the finger after its stimulation.

At the cortical level, RSS of a finger was repeatedly found to induce plastic changes in the representation of this finger within SI and the secondary somatosensory cortex (SII)^8–11,24^. Using magneto-encephalography (MEG) recordings, we recently found additional plastic changes in the lip representation in SI^6^. Together with the perceptual improvement reported at the stimulated finger and face^12^, these MEG data suggest that most of the learning process and transfer may occur in SI. It is commonly accepted that the hand representation in SI is surrounded ventrally by the representation of the lip and dorsally by the representation of the forearm^1,25^. Based on this view our results suggest that the proximity in SI alone cannot explain the cross-border transfer of RSS-induced changes. However, the notion of “cortical proximity” has to be taken with caution since in our case the index finger representation is not strictly adjacent to the lip and forearm representations as the other fingers and palm representations are in between D2 and the forearm, and the thumb and upper face in between D2 and the lips. In addition, SI organization appears to be much more complex than that suggested by the famous Penfield *Homunculus*. For example, a recent study revealed that even for the most studied body part (i.e., the hand), the exact extent and location of its cortical representation is still matter of discussion^26^. In addition, the lack of changes at the forearm seems to be in contrast with the classical view from deprivation studies that neighbouring cortical territories are competing for inputs^3,13,27^. As our findings suggested a lack of plastic changes in the neighbouring forearm representation, we suggest that competitive mechanisms that extend into both sides of the hand representation come into play only when there are reduced inputs; while, other non-competitive mechanisms may control transfer when there are increased inputs.

A possible explanation for this could be that the hand and face functional representations are more strongly linked than those of the hand and the forearm. This could arise from the massive co-use and thus co-activation of these two body parts during very fundamental and automatic behaviours such as feeding^28^. This co-use could result in a higher functional connectivity between the hand and face representations in SI and, thus, favour transfer of plastic changes across the hand-face border. Reciprocally, the physically impossible co-activation of the hand and ipsilateral forearm might result in low functional connectivity between their representations, making transfer of plastic processes more difficult. Evidence supporting the impact of limb use on the transfer of learning comes from a recent study showing that the transfer of learning between fingers is related to differences in finger use^29^.

The higher co-use and co-activation between the hand and the face could also result in specific functional units triggered by ecologically-relevant movements, as previously described in the primary motor cortex (M1, see^14^). While our results point to the possible existence of similar functional units in SI, some intrinsic differences between SI and M1 might limit the analogy. Indeed, while movements are necessarily directed towards a given location or function (e.g., mouth or defensive behaviour), tactile inputs remain marginally constrained to their location on the skin surface. Consequently, the movements we do are likely to affect the probability of tactile co-activation between body parts, and thus their functional connectivity, but SI representations are likely to remain quite stable. In line with this, a study showed that changing the hand posture (mimicking different actions), affects representations in SII but not in SI (Hamada & Suzuki, 2005).

Alternatively, the transfer of plasticity and the resulting learning effects could depend on long-range cortico-cortical connections crossing the hand-face functional border in SI^30–34^. However, the existence and involvement of such connections in large-scale plastic reorganization is controversial. Indeed, some electrophysiological studies reported that these connections are few^30^ and unable to undergo sprouting following chronic unilateral lesions of the dorsal columns^34^, at least within Brodmann area 3b. In contrast, other studies reported large overlaps and strong connections between the hand and lower face representations^32^, and more widespread connections after incomplete vs. complete deafferentation^35^. Closer analysis of the functional and plastic properties of these horizontal circuits revealed that the hand-face border does not restrict plastic changes, and that connections crossing this border may have intrinsic properties that differ from those remaining within a given representation^36,37^. While these latter results strongly support the involvement of such cross-border connections in the transfer of learning we observed from the hand to the face, their existence across the hand-arm border is still unknown. Assuming that hand-arm horizontal connections exist, our results suggest they may be less numerous and/or may not undergo plasticity as easily as those crossing the hand-face border. Further studies are needed to address these questions.

While SI is the plausible origin of the observed transfer of plastic processes, other substrates may also contribute. For instance, it is possible that these plastic changes are measureable in SI but actually originate somewhere else. Since reorganization following RSS was also found in SII^9^, plastic changes could also transfer at this level. However, while SII is known to present a similar topographic organization as SI^38,39^, its neurons tend to have larger and more complex receptive fields^40,41^. In the present case, this would facilitate unspecific transfer of learning to the forearm. In addition, SII is usually involved in processing more complex tactile information such as textures and shapes^42^, attention^43,44^, and working memory of tactile textures^45^, all of which had little involvement in the present experiment. From that perspective, changes in the subcortical areas would be more likely. For instance, the thalamus^46^ or even the cuneate nucleus^47,48^ could be the origin of the plastic changes and transfer of learning that are reflected in SI.

Whatever the substrate, the lack of correlation between the threshold changes observed at the finger and at the lip suggest that the amount of transfer may depend on multiple substrates and/or on the strength of functional connectivity between the cortical representations of these two body parts.

A limitation of this study comes from the fact that the present results were obtained after stimulating the right-index finger. The generalisation to the hand made here is conceptual and based on the functionality of the hand, which allows to consider it as a single functional unit^14^. Further investigation is required to explore the pattern of generalisation after stimulating other parts of the hand. In addition, the present results do not allow to say whether the relationship between hand and face is exclusive or not. Further studies are required to investigate the pattern of generalisation obtained after stimulation of body parts other than the hand. Note that despite the fact that some participants were retested in Experiment 2, the tests were 8 to 9 months apart, making unlikely any maintenance of learning or plastic processes. This is further confirmed by the very similar baseline threshold these participants exhibited in both experiments (27.55 ± 3.57 in Experiment 1 and 27.60 ± 4.63 in Experiment 2).

Altogether, our results provide the first evidence that the plastic processes and tactile improvement induced by RSS transfer to distant body parts in a specific way (i.e., preferentially from the hand to the face rather than to the forearm). This suggests a strong functional coupling between the hand and the face representations, which could result from an evolutionary-based, fundamental co-use due to feeding behaviours.

## Methods

### Participants

Twenty-eight healthy volunteers (mean age: 23.35 years ± 2.51 SD, eight males) were tested. Twenty of them took part in the first experiment and twelve were re-tested in the second experiment after 8 to 9 months, in addition to eight additional participants. All participants were right-handed according to the Edinburgh Handedness Inventory^49^; mean score = 71.76% ± 13.14 SD), except one who had a score < 40 and was excluded from further analysis. All participants provided written informed consent before participating. The protocol was approved by the ethics committee of Bochum (Ethikkommission der medizinischen Fakultät der Ruhr Universität Bochum, Bad Oehnhausen, Reg. No: 76/2014), and was performed in accordance with the Declaration of Helsinki.

### Experimental design

Both experiments took place over two consecutive days. On the first day, participants underwent a practice session to become familiar with the tactile discrimination task before undergoing the first assessment of their tactile spatial acuity, as measured by an improved and modified version of the 2-point discrimination threshold (2PDT). The next day, an additional measure of the 2PDT was acquired before and after 40 min of RSS of the right-D2 (Experiment 1) or of the right-fArm (Experiment 2).

### Repetitive Somatosensory Stimulation (RSS) Protocol

A high frequency mechanical RSS procedure was used^15^. In the first experiment, a small device comprising two movable pins (each having a 0.8 mm diameter) separated by 5 mm^50^ and controlled by a MP3 player was taped to the volar surface of the right-D2 fingertip. For the second experiment, since the optimal distance necessary between the movable pins to induce RSS effects is not known, we choose to adapt this distance individually for the stimulation of the forearm. Two of these devices were taped to the volar surface of the right-fArm of participants centred on the location of 2PDT testing, separated by a distance proportional to the baseline 2PDT of each individual. The factor used to calculate this distance corresponded to the ratio between the pin distance used for the finger stimulation (5 mm) and the baseline 2PDT obtained for the right-D2 in the first experiment. For the participants who took part in both experiments (n = 12), the individual ratio obtained in the first experiment was used to calculate the distance for RSS on the forearm [Distance RSS right-fArm = baseline 2PDT right-fArm x (5/baseline 2PDT right-D2)]. For the eight participants who took part only in the second experiment, the RSS distance on the forearm was calculated using the average ratio obtained for the twenty subjects of the first experiment for the stimulation of right-D2 (mean ratio = 3.00 ± 0.50 SD) [Distance RSS right-fArm = baseline 2PDT right-fArm x mean ratio]. This resulted in an average ratio of 3.03 ± 0.59 (SD), and an average distance between pins/devices of 8.41 mm ± 1.37 (SD) for the stimulation of the forearm. Across the two groups, the average ratio between the RSS distance and baseline 2PDT was 3.02 ± 0.54 (SD). Three participants (one in Experiment 1 and two in Experiment 2) with ratios exceeding this global average ± 2.5 SD were excluded from further analysis, resulting in 19 and 17 participants in Experiments 1 and 2, respectively. Each device delivered brief (10 ms) rectangular pulses of supra-threshold tactile stimuli, organized in 1 s long stimulation trains comprising 20 single pulses (i.e., 20 Hz stimulation) interleaved by 5 s of rest. During the 40 min of the RSS procedures, participants were instructed to continue with their daily activities without paying attention to the device, but to avoid intensive use of their fingers. They were also instructed to continuously listen to their own music or to a background rain sound that we provided to cover the device’s noise.

### Tactile spatial acuity assessment

The 2PDT was measured using two-alternative forced-choice task and force-controlled devices. In the first experiment, the 2PDT was assessed at both index fingertips (left-/right-D2), the right-Lip, and the right-fArm three times before (Practice, S1, and S2) and once after (S3) RSS was applied for 40 min to the right-D2. Within a session, each area was tested in a separate block. For a given participant, the order of blocks was maintained across sessions, but the block order was counterbalanced across participants. In the second experiment, the 2PDT was assessed at the right-fArm three times before (Practice, S1, and S2) and once after (S3) RSS was applied for 40 min to the right-fArm. For each participant, the location of the 2PDT assessments for each tested area was kept constant across sessions [Experiment 1: distance from fingertip: 7.05 ± 1.34 mm and distance from finger edge: 6.86 ± 0.55 mm (mean ± SD); distance from mid-lip: 18.84 ± 4.83 mm (mean ± SD), midway between the upper-lip and the base of the nose; distance from wrist: 8.82 ± 1.89 cm (mean ± SD), on the forearm midline; Experiment 2: distance from wrist: 8.72 ± 1.69 cm (mean ± SD), on the forearm midline].

The 2PDT was assessed using a procedure similar to the one described by Muret and colleagues^6,12^. This procedure corresponds to an improved version of the classical 2-point discrimination task. In this version, the threshold does not correspond to the distinction between 1 tip versus 2 tips, but to the decision when 2 tips are sufficiently separated to be perceived as two. To this aim, the entire psychometric curves were computed and then used to determine the distance at which participants reported the sensation of two clearly separated tips (as compared to two tips less distant, perceived as one tip). In brief, eight probes were used, one with a single tip and seven with two tips separated by various distances. Due to differences in absolute sensitivity, various sets of distances were used for the index fingertips (0.7, 1.0, 1.3, 1.6, 1.9, 2.2, 2.5 mm), the lip (2, 3, 4, 5, 6, 7, 8 mm), and the forearm (10, 15, 20, 25, 30, 40, 50 mm). After allowing the participant to feel the extreme separation distances three times to make sure they clearly felt the difference between the two tips, the testing began. Participants were instructed to relax and to remain still. The probes were brought into contact with the participant’s skin for approximately 1 s and they were required to promptly say whether they felt “one” or “two” probes. Emphasis was laid on answering “two” only when clearly perceiving two distinct points. When perceiving a bar, a bigger point or any unclear shape, participants were instructed to answer “one”. Accuracy was encouraged over speed, with no time limit being imposed. Each probe was tested eight times in a pseudo-randomized order, resulting in 64 trials per body part. Tips were always presented parallel to the longitudinal axis of the fingers, face, and forearm. Two specially-designed spring-mounted apparatuses were used to ensure almost-constant application force across trials (see^12^ supplemental data for details).

### Data and statistical analyses

For each participant, body part, and session, the mean of the verbal responses “two” was plotted as a function of distance between the probes and the psychometric function was fitted using a binary logistic regression. The threshold was determined from the fitted data and defined as the distance at which participants responded “two” 50% of the time. Two participants (one in each experiment), who showed very unstable baselines (i.e., S1 and S2 threshold differences exceeding the mean ± 2.5 SD), were excluded from further analyses. The S1 and S2 thresholds were statistically analysed for stability using paired t-tests. Pre-(average of S1 and S2) and post-(S3) thresholds were then analysed using paired t-tests. The level of significance was Bonferroni-corrected for the multiple tests (*p*_*Bonf*_ = 0.01). To test for changes in the discrimination sensitivity and response criterion (Signal Detection Theory), false alarm and hit rates were calculated and used to derive the discriminative index (d-prime value) and the criterion shift index (ln(Beta) value) using the MATLAB Palamedes toolbox for one-alternative forced choice (PAL_SDT_1AFC_PHFtoDP, MATLAB r2016a). Statistical analysis was performed using paired t-tests with a Bonferroni-corrected threshold of *p*_*Bonf*_ = 0.01. Effect sizes were calculated using the Cohen’s d coefficient.

To compare RSS-induced changes across regions, threshold changes between pre- and post-sessions were expressed as a percentage of the pre-session threshold for each individual. These values were then submitted to five t-tests against zero to confirm significance, with a Bonferroni-corrected level of significance (*p*_*Bonf*_ = 0.01). In the first experiment, a repeated measure analysis of variance (ANOVA) was then used to compare these values across regions, followed by Bonferroni post-hoc analysis. A linear correlation was also performed to compare threshold changes obtained at the right-D2 and right-Lip. In the second experiment, an independent t-test was used to compare threshold changes obtained at the right-fArm in both experiments. All statistical analyses were conducted using STATISTICA^®^ (v.12, StatSoft). Data were checked for normality using Kolmogorov-Smirnov tests. All group data are expressed as the mean ± standard error of the mean (SEM).

### Data availability

The datasets generated and analysed during the current study are available from the corresponding author on reasonable request.

## Electronic supplementary material

Supplementary information
