# Supplementary material for: Tactile learning transfer from the hand to the face but not to the forearm implies a special hand-face relationship
Source: Sci Rep. 2018 Aug 6;8:11752. doi: 10.1038/s41598-018-30183-5 (PMC6079060; doi:10.1038/s41598-018-30183-5)
Supplement: Supplementary file 1 — Supplementary information [file 41598_2018_30183_MOESM1_ESM.pdf]

Supplementary Information

Tactile learning transfer from the hand to the face but not to the forearm implies a special hand-face relationship

Dollyane Muret[1,2*] & Hubert R. Dinse[1,2]

Body part	Right-D2		Left-D2		Right-Lip		Right-fArm	
Subject	Pre	Post	Pre	Post	Pre	Post	Pre	Post
1	2.00	1.80	1.91	2.16	7.07	6.49	33.21	28.38
2	1.45	0.98	1.27	1.22	4.31	2.93	18.75	16.83
3	1.60	1.33	1.47	1.53	5.19	5.37	36.48	39.95
4	1.62	1.59	1.75	1.98	5.87	5.50	26.65	27.37
5	1.63	1.71	1.70	1.63	5.67	5.53	26.29	28.94
6	1.73	1.56	1.81	1.75	7.05	6.63	30.64	35.16
7	1.78	1.68	1.92	1.90	5.62	5.76	26.92	28.26
8	1.64	1.56	1.69	1.76	5.44	4.60	27.91	27.60
9	1.73	1.71	1.54	1.59	6.00	5.76	32.37	34.17
10	1.94	1.76	1.37	1.37	6.07	5.13	29.47	28.61
11	2.11	2.09	1.88	2.06	6.17	5.37	31.33	31.00
12	1.88	1.67	1.86	2.05	6.33	6.37	30.58	30.58
13	1.79	1.52	1.71	1.49	5.69	5.89	23.46	25.49
14	1.82	1.71	2.05	1.67	5.63	4.91	28.22	25.62
15	1.52	1.44	1.48	1.37	5.13	4.37	30.51	27.13
16	1.59	1.60	1.58	1.64	5.00	5.00	21.90	26.74
17	1.88	1.98	1.81	1.89	5.93	5.53	32.39	28.60
18	1.71	1.56	1.60	1.56	6.15	4.61	35.08	37.94

Supplementary Table S1. Thresholds of individuals who took part in Experiment 1, Pre and Post RSS on the right-D2.

Body part	Right-fArm	
Subject	Pre	Post
1	31.30	29.74
2	21.26	20.65
3	26.76	23.47
4	23.85	22.62
5	25.01	23.36
6	29.25	27.14
7	29.76	29.21
8	29.11	29.21
9	31.68	33.65
10	27.23	24.97
11	33.04	28.15
12	26.01	23.58
13	28.50	28.15
14	30.18	27.14
15	31.10	33.07
16	25.13	23.17

Supplementary Table S2. Thresholds of individuals who took part in Experiment 2, Pre and Post RSS on the right-fArm.

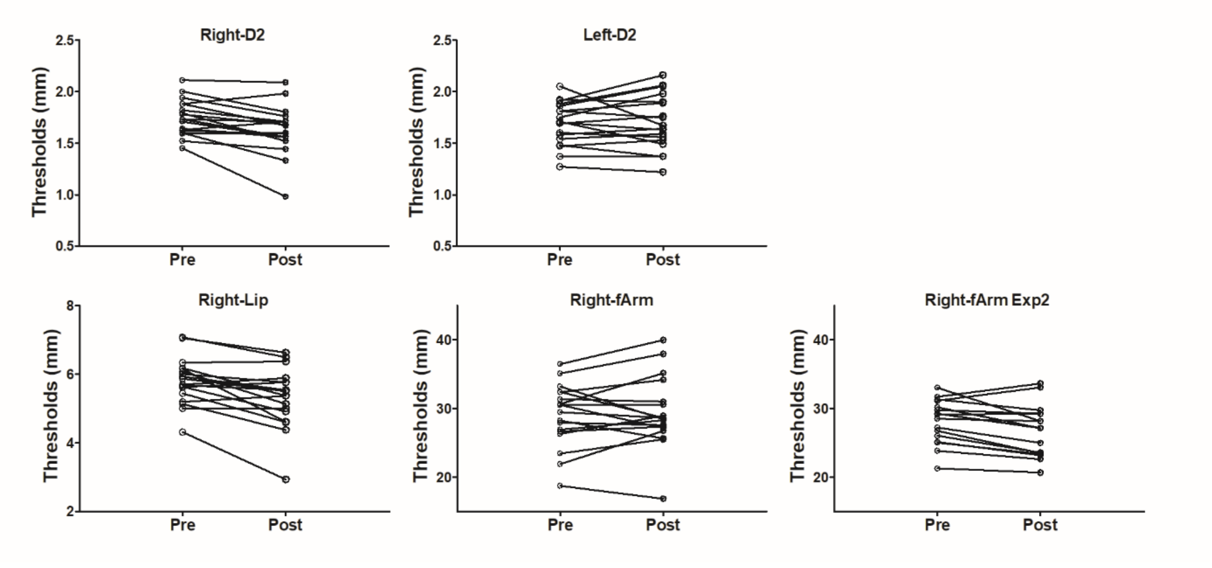

Supplementary Figure S1. Thresholds (in mm) of individuals obtained on the right-D2, left-D2, right-Lip and right-fArm in Experiment 1 (Pre and Post RSS on the right-D2), and obtained on the right-fArm in Experiment 2 (Pre and Post RSS on the right-fArm).

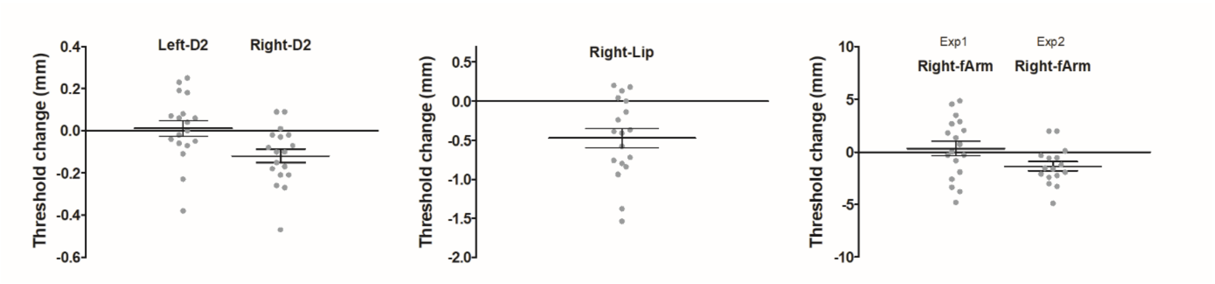

Supplementary Figure S2. Threshold changes (in mm) of individuals obtained on the left-D2, right-D2, right-Lip and right-fArm in Experiment 1 (RSS on the right-D2), and obtained on the right-fArm in Experiment 2 (RSS on the right-fArm).
